# The impact of human cadaveric dissection on professional identity formation in medical students

**DOI:** 10.1186/s12909-023-04913-x

**Published:** 2023-12-19

**Authors:** Ci Xin Ong, Yang Yann Foo, Scott Compton

**Affiliations:** Duke-NUS Medical School, 8 College Rd, Singapore, 169857 Singapore

**Keywords:** Human cadaveric dissection, Professional identity formation, Medical students

## Abstract

**Background:**

As technology advances, some schools are moving away from human cadaveric dissection to teach anatomy, leading to concern regarding the possible loss of a professional identity building experience. This study explored the role of dissection in students’ professional identity formation.

**Methods:**

A mixed-methods study was conducted using survey methodology and semi-structured interviews of medical students at an American-style graduate-entry medical school in Singapore. The questionnaire adopted the conceptual framework of the Ring Theory of Personhood and the MacLeod-Clark Professional Identity Scale was used to measure professional identity, followed by semi-structured interviews of students using Braun and Clarke’s six-phase reflexive thematic analysis.

**Results:**

Respondents did not differ substantively from non-respondents by age, nationality, or ethnicity, and year of entering medical school, however, they were slightly more female dominant. The number of hours of hands-on participation in dissection showed no significant relationship (r^2^ = 0.010; *p* = 0.424) with professional identity formation measured by the MacLeod-Clark Professional Identity Scale. Despite the survey results, semi-structured interviews revealed rich and nuanced findings suggesting the influence of dissection in participants’ professional identity formation through deepening students’ appreciation of humanistic values and enhancing their notions of patients’ personhood. Notably, students without dissection experience did not express these sentiments and were orientated towards knowledge acquisition.

**Conclusion:**

While our findings do not suggest that dissection strongly impacts students’ professional identity formation, students shared thought-provoking experiences which suggest some level of its contribution. Careful consideration of this phenomenon should be exercised prior to removing dissection in favour of technological alternatives.

**Supplementary Information:**

The online version contains supplementary material available at 10.1186/s12909-023-04913-x.

## Background

Human cadaveric dissection has historically been considered an essential educational tool to teach anatomical science [1]. Despite being recognized as a key experience in medical education [2], the number of hours dedicated to dissection classes has significantly declined over the years [3], and in a few institutions, it has even been removed entirely from the curriculum [4–6]. The debate about whether or not to use dissection as an important teaching method in medical education continues. It has been further highlighted by the Coronavirus disease 2019 (Covid-19) pandemic when many traditional physical anatomy classes were required to be delivered via online methods across various institutions [7–9]. This growing trend of medical schools pivoting away from dissection may have serious implications for professional identity formation.

Professional identity formation (PIF) is a multifaceted, individualized process of the transformation of a lay person into a physician [10] conceptualized by the development in which an individual identifies themselves as a part of the profession, through the acquisition of essential knowledge, skills, attitudes, values, and behaviour [11–13]. In a study by Monrouxe, Rees and Hu [14], it was found that early exposure to patient interactions and participation in small group discussions led by clinicians contribute significantly to students’ development of professional identity as engaging in interactive and meaningful activities within the formal professional curriculum encourages students to have a deeper understanding of intricate human experiences. The dissection course as part of the medical school’s curriculum has the potential to broaden the range of learning outcomes associated with essential skills and attitudes in the process of PIF [15].

Evidence suggests that dissection is an effective method of learning human anatomy as it improves examination scores [16–18] and enhances students’ motivation in learning anatomical science [19]. Since the emergence of modern alternative teaching methods, there has been an increased focus on evaluating the efficiency and applicability of the traditional method of teaching anatomy in contrast to these newer technological options. For instance, a meta-analysis of anatomy laboratory pedagogies showed no statistical significance of student performance scores when comparing traditional dissection to other laboratory methods (such as prosection, digital media, 3D models/modelling, and hybrid approaches) [20]. However, newer three-dimensional anatomy visualization methods (i.e., virtual reality, augmented reality, and computer-based three-dimensional visualizations) were overall more effective in promoting anatomical knowledge compared to traditional methods that have been used for decades (i.e., cadaver and textbooks) [21].

While there may be advantages to acquiring knowledge using other teaching modalities than dissection, there is much to be known about the impact towards developing the soft skills of a future physician. Dissection could also play an important role in PIF through reinforcing compassion and respectful attitudes [22, 23] and patient-centered professionalism [24] as seen in various qualitative research using oral [25] and written reflections [2, 25] as well as surveys questionnaire [26]. Interestingly, a study also showed students perceived plastinated specimens with more respect and care than three-dimensional printed models as they recognised the specimens as more real and authentic [27]. In this manner, dissection providing the real hands-on experience with the human body can be useful in enhancing students’ appreciation and respect, which are part of the process of PIF.

Hence, the possibility of removing dissection from the medical education curriculum has caused some to be concerned that there will be concomitant loss in the development of students’ professional identity as a physician. As a result, over the years, medical schools have developed and implemented a myriad of educational interventions derived from varying interpretations of PIF [28] with the intention to influence the development of a student’s professional identity positively.

Although there is no unified theoretical framework to examine the process of PIF in current literature [29], demographics, values, goals, learning environment, mentors and role models are considered factors which may influence PIF [30–32] and help in understanding the complexities of both the experience and the active constructive process of professional formation [30]. Therefore, the purpose of this study was to investigate the association between the dissection experience in medical school and the degree to which medical students exhibit PIF, while controlling for important variables such as demographic factors, motivation, empathy, mentoring, and learning environment. We further explored medical students’ perceptions of the impact that dissection had on their PIF.

At the time of study, there was a cohort of students affected by the Covid-19 pandemic and learnt human anatomy without the dissection experience. This provided our study with a unique opportunity to explore the differences between students with and without the dissection experience.

## Methods

### Study design

A mixed-methods study was conducted from November 2022 to January 2023 and included a quantitative survey-based component as well as semi-structured follow-up qualitative interviews.

### Conceptual framework

**Fig. 1:**
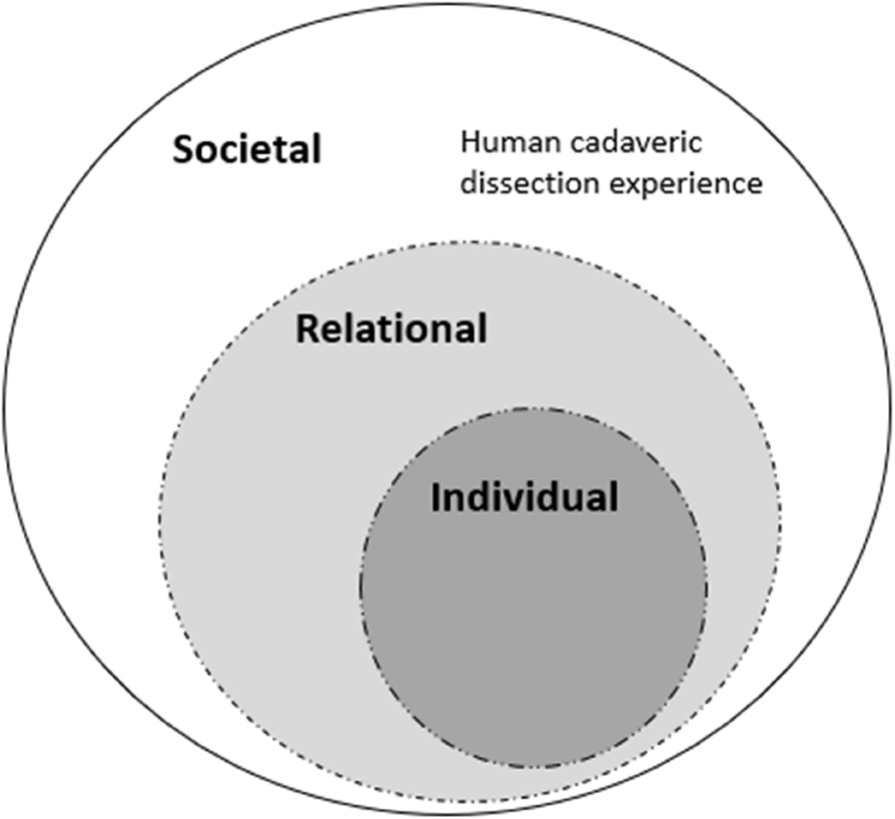
Dissection experience in the Societal Ring of Ring Theory of Personhood framework

We adopted Krishna and Alsuwaigh’s person-centric lens of Ring Theory of Personhood (RToP) [33], which categorizes the influencing factors of PIF in its Individual, Relational and Societal Rings. The Individual Ring represents an individual’s personal emotions and motivation, enclosed by the Relational Ring representing relationships such as supportive clinical interactions between students and doctors and the most outer ring, Societal Ring, includes the learning environment and formal curriculum of a medical education [34]. The dissection experience as part of the medical school curriculum was included in the Societal Ring of the RToP framework as shown in Fig. 1. Through the survey questionnaire and interviews, we sought to understand how these factors and dissection experience could contribute to the process of PIF.

### Participants & setting

Eligible participants were currently enrolled medical students at the Duke-NUS Medical School, an American-style, four-year graduate-entry medical school located in the Republic of Singapore. It is a partnership between the Duke School of Medicine (Durham, NC) and the National University of Singapore.

Traditionally, Duke-NUS Medical School has included 15 three-hour cadaver sessions over 5 months as part of its first-year course covering human anatomy. However, due to the Covid-19 pandemic and resulting social distancing requirements and difficulty procuring human cadavers, students in the entering cohort of 2020 were not offered a dissection experience. For these students, anatomy was taught through other resources including textbooks and various digital resources. The school resumed the dissection course for the subsequent cohorts, thus, of the four cohorts enrolled at the time of this study, three (Years 1, 2 and 4) had been exposed to cadaveric instruction and one (Year 3) was not.

A single cadaver would be allocated to a group of 7 to 8 students in the dissection course. Students would take turns to participate in hands-on dissection voluntarily. Hence, there may be variations in the number of hours of dissection experience for every student.

### Survey development and dissemination

We developed an online questionnaire inspired from the RToP framework, to assess quantitatively the eight domains including demographics, level of intrinsic and extrinsic motivation and awareness of emotions such as empathy, student’s relationship with their mentors and their perspective of the learning environment, as well as their professional identity and dissection experience as shown in Table 1. A 46-item questionnaire was created by amalgamating items from various previously validated questionnaires on motivation, empathy, learning environment and professional identity. Questions on demographics, mentors, and the number of hours of observed and hands-on participation in dissection experience were also included in the questionnaire. The psychometric properties of the scale items are shown in Table 2, along with the reference and their previously reported reliability and internal consistency as calculated by Cronbach’s coefficient alpha. The full 46-item questionnaire can be found in Additional file 1.

**Table 1:** Survey domains inspired by the Ring Theory of Personhood (RToP) framework

Ring Theory of Personhood	Survey Domains
Individual Ring	Intrinsic Motivation
	Extrinsic Motivation
	Empathy
Relational Ring	Student-Mentor Relationship
Societal Ring	Dissection experience
	Learning Environment
	Demographics
	Professional Identity

**Table 2:** Calculated reliability statistics of survey instruments and comparison with previously reported values

Scale	Number of Items	Obtained Cronbach’s Alpha	Previously reported Alpha	References
Demographics	6	Not applicable	Not applicable	–
Intrinsic Motivation	4	0.694	0.79 [35]	Motivated Strategies for Learning Questionnaire (MSLQ) [36]
Extrinsic Motivation	4	0.759	0.78 [35]	Motivated Strategies for Learning Questionnaire (MSLQ) [36]
Empathy	8	0.664	0.7 to 0.8 [37]	Interpersonal Reactivity Index (IRI) [38]
Dissection experience	2	Not applicable	Not applicable	–
Mentor	2	Not applicable	Not applicable	–
Learning Environment	8	0.763	0.94 [39] to 0.97 [40]	AAMC Graduation Questionnaire (AAMC-GQ) [41, 42]
Professional Identity	9	0.903	0.83 [43]	MacLeod Clark Professional Identity Scale (MCPIS-9) [44]

An email invitation was sent to all eligible students with a participation information sheet and a link to complete the 10-minute self-administered questionnaire anonymously. At the end of the online survey, students who were willing to participate in a zoom interview were asked to provide their consent for future follow-up. They could access a separate link where they could leave their name, email, age, gender, current year of study and answer a yes-no question to dissection experience after consent was given. There was no linkage between the survey responses and contact information. A reminder email was sent a week later to eligible students. This study was approved by the National University of Singapore Institutional Review Board (Ref: DERC-02-221020).

A $5 voucher to a local food delivery service was available to students as reimbursement for completion of survey. Instructions were given to take a screenshot and email the final page of the survey to the school’s department of education. The results of the survey were kept anonymous. An additional $15 voucher was provided to the 12 students who participated in a follow-up interview.

### Survey data

We report descriptive statistics of all demographic data, dissection experience, and construct measures obtained from the survey. We then obtained bivariate correlations of each of those variables with the primary outcome of “professional identity score”.

All survey data was analysed using SPSS version 26 (IBM Corp, Armonk, NY, USA).

### Qualitative data collection methods

To provide deeper insight into the role of dissection in PIF, we followed up the survey with in-depth semi-structured interviews of a selected subset of students (*n* = 12), to better understand the respondents’ experience in dissection course and explore their perception of how the experience could impact their PIF as a doctor. The interviews were all conducted in-person by one interviewer (OCX) and each took between 15 to 30 minutes.

Out of the 69 respondents of the online survey, 42 (61%) had indicated interest in participating in a follow-up interview. Purposive quota sampling strategy was implemented to ensure the same number of respondents were selected across all 4 years. Three respondents from each cohort, Years 1 to 4, were interviewed. Six males and six females were interviewed, with one male and two females from the non-dissection cohort (Year 3), and five males and four females from the dissection cohorts (Years 1, 2 and 4). The interviewer used a semi-structured topic guide, and all the interviews were conducted in English over a six-week period between December 2022 to January 2023. Informed consent was obtained at the end of the online survey and verbal consent at the start of the interview. Permission was sought for the interviews to be audio-recorded for transcription purposes. All interviews were transcribed verbatim by OCX.

### Reflexivity [45]

The interviewer (OCX) is presently enrolled as a medical student at Duke-NUS and belongs to the 2020 entering cohort who went through the first-year curriculum without the dissection course because of the Covid-19 pandemic. OCX was trained to develop the topic guide, and iteratively collected and analysed data to refine the topic guide by FYY, a qualitative researcher and reflexive thematic analysis practitioner at the Academic Medicine Education Institute, which is affiliated with Duke-NUS Medical School.

### Qualitative data analysis

OCX and FYY analysed the interview transcripts using Braun and Clarke’s six-phase reflexive thematic analysis (RTA) informed by a critical realist ontology which asserts that reality is accessible but is mediated by sociocultural meanings [46]. OCX and FYY independently wrote familiarization notes (phase 1) and coding (phase 2) was conducted inductively (at latent and semantic levels) and deductively (sensitized by RToP constructs) for two transcripts, one of a participant with dissection experience and one without. Discussions followed where they examined the content of the codes, focusing on whether and how dissection influenced PIF. OCX subsequently coded the remaining 10 transcripts and, at regular meetings with FYY, constructed candidates themes (phase 3) through the use of clustering and code-promotion techniques [46]. Using thematic mapping and the principles of central organizing concepts, the candidate themes were refined (phase 4) and finalized (phase 5). OCX wrote the manuscript (phase 6) with inputs from FYY.

## Results

### Survey results

Overall, 69 of 301 eligible students responded to the online survey, yielding a response rate of 23%. Out of the 69 respondents, there were 16 respondents from Year 1, 17 respondents from Year 2, 20 respondents from Year 3, and 16 respondents from Year 4. Respondents did not differ substantively from non-respondents by age, nationality, or ethnicity, and year of entering medical school, however, were slightly more female dominant.

**Fig. 2:**
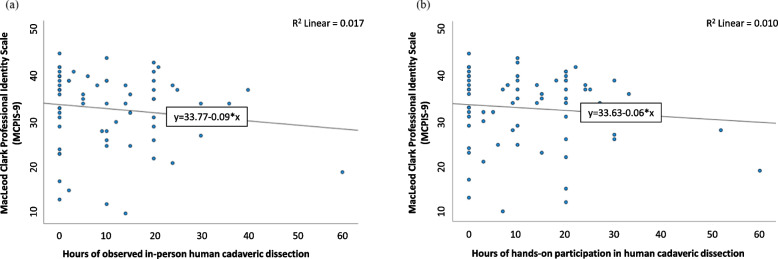
Professional identity scores of respondents by the number of hours of (**a**) dissection observation experience, and (**b**) hands-on dissection experience

The number of hours of observed (r^2^ = 0.017; *p* = 0.286) and hands-on (r^2^ = 0.010; *p* = 0.424) participation in dissection showed no significant relationship with PIF measured by the MCPIS-9 in Fig. 2a and b respectively. We also explored the bivariate relationship between PIF and all survey factors such as demographics, level of motivation and empathy, relationship with mentors, students’ perspective of the learning environment, observed and hands-on dissection experience. From that analysis, female gender and students’ perspective of the learning environment were statistically associated with the PIF score, as shown in Tables 3 and 4.

**Table 3:** Demographic characteristics of respondents and professional identity score

	Mean Professional Identity Score (*n* = 69)	Correlation with Professional Identity Score (*p*-val)
Gender, n (%)		
Male, 25 (36.2%)	29.5 (9.0)	0.311 (0.009)
Female, 44 (63.8%)	34.7 (7.1)	
Age, n (%)		
21–24 years, 12 (17.3%)	34.3 (10.1)	−0.125 (0.307)
25–29 years, 41 (59.4%)	33.0 (7.7)	
> 30 years, 16 (23.2%)	31.2 (8.1)	
Year in Medical School, n (%)		
I, 16 (23.2%)	32.5 (9.7)	−0.086 (0.482)
II, 17 (24.6%)	34.3 (6.8)	
III, 20 (29.0%)	33.6 (8.6)	
>IV, 16 (23.2%)	30.6 (7.4)	
Physician family member, n (%)		
Yes, 10 (14.5%)	34.1 (10.1)	0.064 (0.602)
No, 59 (85.5%)	23.6 (7.9)

**Table 4:** Description of study variables and correlation with professional identity score

	Mean (SD) n = 69	Correlation with Professional Identity Score (*p*-val)
Hours of Observed Dissection	10.8 (12.0)	−0.130 (0.286)
Hours of Hands-On Dissection	12.6 (12.3)	−0.098 (0.424)
Intrinsic Motivation^a^	22.0 (3.2)	0.063 (0.608)
Extrinsic Motivation^a^	18.4 (4.7)	−0.057 (0.642)
Learning Environment	33.8 (5.0)	0.285 (0.018)
Empathy	31.6 (3.7)	0.024 (0.844)
Mentor	4.9 (1.6)	−0.029 (0.815)

^a^ *n* = 68; One student did not complete the MSLQ Intrinsic and Extrinsic Motivation items on the questionnaire

### Qualitative findings

While the survey did not show statistical significance, the qualitative part of the study revealed rich and nuanced findings showing how dissection influenced participants’ PIF. Most students shared how dissection seemed to have deepened their sense of humanistic values and enhanced their notions of patients’ personhood. By comparison, non-dissection students talked about anatomy almost exclusively from the perspective of knowledge acquisition.

#### Theme 1: Dissection deepens students’ appreciation of humanistic values

Dissection appeared to evoke strong emotions in participants which deepened their understanding of humanistic values such as compassion, empathy, and respect for others. Many of these participants reported feeling “emotional” (P4; Year1 medical student) when learning anatomy through dissection. This was especially so for their first dissection class as participants did not have prior contact with a cadaver and the experience seemed to be highly visceral:

I actually had a panic attack, you know, as in, umm I was really very uncomfortable with, you know, cutting open a human body. (P3; Year4 medical student)

The journey of learning anatomy through dissection was an intensely emotional one as students had to cut “through all the layers” of skin, muscles, and tissues to get to organs such as “the heart” (P2; Year2 medical student). Notably, many students shared how they were taught by the faculty to handle the process in a way “to show the cadavers the respect that they deserve since they volunteered their bodies for science” (P8; Year1 medical student). They were humbled by the donors’ noble sacrifice to enhance the medical education of future physicians:

We always believe that when we die, we have to have the full body and then just let it decay in the coffin, not being touched by other people, or take out their organs or something, but they are willing to contribute their body. (P5; Year2 medical student)

The gratitude the students felt towards the selfless individuals who donated their bodies to medical education also helped the students to feel “a lot of compassion” (P7; Year4 medical student) and “empathy and the respect for life” (P11; Year1 medical student). The sense of appreciation was further highlighted on the Donor Memorial Day, an annual tradition of the school where participants conveyed their gratitude through hand-written notes and poems. They were encouraged to reflect deeply on the significance of their authentic learning opportunity to thank their cadavers as their silent teachers:

We wrote cards, we wrote poems…to kind of express… “You’re like our teacher as well, and you know thank you really thank you for that opportunity to learn from you”. (P3; Year4 medical student)

The dissection experience was an unforgettable experience for many participants as they learnt to appreciate the donors’ sacrifice. The process seemed to have deepened their humanistic values such as respect, empathy, and compassion.

#### Theme 2: Dissection enhanced students’ notions of patients’ personhood

By learning about anatomy through dissection, participants shared how they started to notice social aspects associated with the human body. They could see their cadaver who “was once a person who they have their own life, their own dreams, their own aspirations” (P12; Year2 medical student). This emphasized the concept of personhood as participants recognized their cadaver as a person who had lived, loved, and lost, and that their patient’s disease was just a part of their identity:

Our patient’s liver was quite cirrhotic and then I thought, oh, this was because our patient drank alcohol, and then I started to think of all the memories, the patient or the cadaver might have. (P7; Year4 medical student)

Having a heightened sense of patients’ personhood seemed to have the effect of encouraging students to regard patients as individuals. Several spoke of how when they become physicians, they would “not just purely treat the (patient’s) disease” (P5; Year2 medical student) but also consider other areas such as the social determinants of health:

I think it is the aspect of building that sense of empathy and seeing what’s beyond the patient’s presentation… and look at what are some of the social factors that we could improve. (P9; Year4 medical student)

Thus, dissection experience seemed to provide a precious window through which medical students could see patients as unique individuals to whom they would provide care in a more compassionate and caring way.

#### Theme 3: Learning anatomy without dissection orientated students towards knowledge acquisition

Participants without dissection experience reported knowledge acquisition as the main purpose in learning anatomy. They did not express the same sentiments as shown in the previous two themes by students with dissection experience. For instance, they did not talk about patients’ personhood and relating to them as individuals. The focal point for learning anatomy seemed to be all about medical knowledge. When asked, “Apart from knowledge, how do you think learning from non-dissection resources has contributed to your development in becoming a doctor?”, participant 1 responded, “Honestly, I don’t think (it did) so.” He saw it as simply a tool to learn human anatomy which could be easily substituted by other learning resources:

If you really are interested in anatomy, you will find your own ways to learn it. I don’t think really need to rely too heavily on cadaveric dissections. (P1; Year3 medical student)

Another participant without dissection experience shared how she would use her anatomical knowledge in relation to her patient, which was primarily confined to scientific knowledge and facts:

If patient asks any questions and if it is somewhat connected to anatomy, then I can try to answer. (P10; Year3 medical student)

However, when the focal point was solely on factual anatomical knowledge, there seemed to be little consideration given to physician-patient relationships. Lack of dissection experience appeared to have deprived students the opportunity to engage in emotions such as empathy, respect, and compassion in relation to patients, which are key aspects of PIF.

The 3 study themes are summarized with representative quotes in Table 5.

**Table 5:** Summary table of study themes with representative quotes

**Theme 1: Dissection deepens students’ appreciation of humanistic values.** The gratitude the students felt towards the selfless individuals who donated their bodies to medical education helped the students to feel compassion, empathy and the respect for life.	
The journey of learning anatomy through dissection was an intensely emotional experience.	*I actually had a panic attack, you know, as in, umm I was really very uncomfortable with, you know, cutting open a human body. (P3; Year4 medical student)*
Students were humbled by the donors’ noble sacrifice to enhance the medical education of future physicians.	*We always believe that when we die, we have to have the full body and then just let it decay in the coffin, not being touched by other people, or take out their organs or something, but they are willing to contribute their body. (P5; Year2 medical student)*
They were encouraged to reflect deeply on the significance of their authentic learning opportunity to thank their cadavers as their silent teachers.	*We wrote cards, we wrote poems…to kind of express… “You’re like our teacher as well, and you know thank you really thank you for that opportunity to learn from you”. (P3; Year4 medical student)*
**Theme 2: Dissection enhanced students’ notions of patients’ personhood.** The dissection experience seemed to provide a precious window through which medical students could see patients as unique individuals to whom they would provide care in a more compassionate and caring way.	
The concept of personhood was emphasized as participants recognized their cadaver as a person who had lived, loved, and lost.	*Our patient’s liver was quite cirrhotic and then I thought, oh, this was because our patient drank alcohol, and then I started to think of all the memories, the patient or the cadaver might have. (P7; Year4 medical student)*
Participants expressed their intention that when they become physicians, their approach would extend beyond merely addressing the patient’s disease; they would also consider various aspects of a patient’s well-being, including the social determinants of health.	*I think it is the aspect of building that sense of empathy and seeing what’s beyond the patient’s presentation… and look at what are some of the social factors that we could improve. (P9; Year4 medical student)*
**Theme 3: Learning anatomy without dissection orientated students towards knowledge acquisition.** Participants without dissection experience reported knowledge acquisition as the main purpose in learning anatomy.	
Participants who did not undergo dissection saw it as simply a tool to learn human anatomy which could be easily substituted by other learning resources.	*If you really are interested in anatomy, you will find your own ways to learn it. I don’t think really need to rely too heavily on cadaveric dissections. (P1; Year3 medical student)*
A participant shared how she would use her anatomical knowledge in relation to her patient, which was primarily confined to scientific knowledge and facts.	*If patient asks any questions and if it is somewhat connected to anatomy, then I can try to answer. (P10; Year3 medical student)*

## Discussion

The purpose of this study was to investigate the association between the dissection experience and the PIF, while controlling for important variables such as demographic factors, motivation, empathy, mentoring, learning environment. The survey results of this study do not suggest that dissection experience strongly impacts students’ PIF. This finding is unexpected as previous studies had shown the student-cadaver relationship as a positive influence in students’ PIF [2, 47, 48] and dissection provided the opportunity to develop social skills, encouraging discussion, and introspection, all of which can shape a student’s professional identity [49, 50]. In addition, in our results shown in Table 3, increasing years in medical school did not correlate with increasing PIF scores. Exemplary role models, effective mentorship, and a nurturing learning environment in medical schools are considered to be the primary factors in driving the process of PIF [51, 52]. Hence, it would be hypothesized for medical students to have greater PIF score as they progress to senior years compared to their juniors. It is surprising that our survey results did not support these hypotheses.

However, upon qualitatively exploring participants’ perceptions of the impact that dissection had on their PIF, students expressed that dissection deepened their sense of humanistic values and enhanced their notions of patients’ personhood. Their perceptions revealed in this study corroborate the important role of dissection in influencing the maturation of moral and humanistic concepts consistent with prior studies [53–55]. These apparent discordant findings between quantitative and qualitative approaches may suggest that the measure of PIF used in this study was not sensitive enough to capture the nuances of the phenomenon.

In relation to the appreciation of humanistic values, there was a study by Weeks, Harris and Kinzey [22] in which four strategies were proposed to instil respect and compassion in medical students through the dissection experience. Firstly, they advocate for the use of the term “donor” instead of “cadaver” to deepen their appreciation for the donated body as the students’ first “patient.” Secondly, instructors should provide personal information about donors, such as their name, age, history, and likely cause of death. Thirdly, students should be encouraged to reflect on their emotions and engage in discussions after the dissection experience. Finally, the authors recommend holding a memorial ceremony involving both students and faculty to provide a closure to the dissection experience, reinforcing positive values and promoting understanding of diverse cultural and religious beliefs. Notably, our school similarly integrates these four strategies into our curriculum and this likely contributed to enhancing students’ recognition and appreciation of humanistic values. The use of these strategies in the dissection course may reduce the tendency towards objectification in the medical profession through humanizing the experience of anatomical dissection.

For several decades, students were taught to focus on acquiring scientific knowledge and ignore the art — the human face — of medicine in cadaveric dissection [56]. However, this emotional detachment towards cadavers has been changing in many institutions [57] which is to give greater attention to the cadaver and future patient as a person rather than a disease [47, 58] so to nurture students as humane physicians. This could be an achievable goal as learning the anatomy with a cadaver is fundamentally an ambiguous encounter; the cadaver was seen as both a human referent and as a learning tool [49, 59]. In this circumstance, it provides an opportunity for students to recognise their cadaver as a person. When a participant saw the cirrhotic liver in her cadaver, she was triggered to think about the cadaver or the patient as a person, and the memories that he or she may hold. The intricacies of the human body serve as a reminder of life. Participants also engaged in reflection from a humanistic perspective, recognizing that people come from diverse backgrounds and are shaped by various social influences, as fully-fledged human beings with their own differences.

Hence, the dissection experience could potentially enhance students’ notions of patients’ personhood and produce physician who are more compassionate and caring. In fact, recent studies also showed patients desiring a positive interpersonal relationship with their doctor, wanting the doctor to treat and care for them as a unique individual [60, 61]. Consequently, a strong physician-patient relationship could increase patient’s medical adherence [62–64] and achieve better results associated with their health [65, 66]. Beyond improving patient outcomes, relational skills improve the patients’ perception of their physicians [67], which reciprocally reinforces the feeling of connectedness and trust between the patient and physicians.

In contrast, participants without dissection experience were orientated towards knowledge acquisition. It was evident in the participants’ responses when asked about learning from non-dissection resources. A participant viewed knowledge acquisition as the sole contribution from non-dissection resources in his development to becoming a doctor whereas the other participants were also focused on factual anatomical knowledge. There was no commentary on developing humanistic values or understanding patients’ personhood from participants without dissection experience. These participants were likely unaware of the limitations in their experience with non-dissection resources. Therefore, the lack of dissection experience appeared to have deprived students the opportunity to engage in emotions such as empathy, respect and in recognising their cadaver or the patient as a person as seen in the previous themes. This was a major distinction between students with and without dissection experience elicited from our study.

In the context of medical education involving the use of dissection, numerous studies had already suggested the educational benefits of developing important humanistic qualities and contributions towards students’ PIF [2, 25, 68–71]. However, our study stands out by demonstrating a clear distinction between the two groups of participants in terms of their key takeaways. The significance of perceiving patients as individuals is a crucial aspect of medical practice considering that patient’s dissatisfaction in modern day medicine rarely stems from the inadequacies of scientific knowledge [72]. Instead, patients are increasingly concerned on how well they could connect and communicate with their physician [73, 74]. Students may find it easier to establish this connection if they possess humanistic values and treat their patients as individuals with unique experiences and feelings beyond their medical condition. This holds significant implications for medical schools looking to adjust their current teaching methods to the learning experience of anatomy.

### Limitations and future research directions

The generalization of the survey results could be compromised as this study used a convenience sample, was conducted at a single institution, and obtained a low response rate. Although results may not represent all medical students everywhere, it provides important methodological considerations for future studies on the topic, most notably in the measurement of the outcome (PIF) and the objective assessment of dissection experience. Future studies may benefit from more precise measures of these constructs.

Given that presently, there is no unified theoretical framework to examine the process of PIF, future researchers may consider collecting more qualitative data from different medical schools using methodologies such as constructive grounded theory to develop from grounds up a theory about how dissection influences PIF.

## Conclusion

This study expands on the possible impact of dissection in PIF. Besides focusing on the score of PIF (whether it increases or not), this study proposes to be cognizant of the unique experience of dissection in providing the opportunity to nurture students in becoming humane and compassionate physicians. Careful consideration of this phenomenon should be exercised prior to removing dissection in favour of technological alternatives.

### Supplementary Information

**Additional file 1.** Survey Questionnaire

## Data Availability

All data generated or analysed from the survey results during this study are included in this published article. There is no public availability to the interview transcripts outside of the research team due to reasons of confidentiality.

## Abbreviations

PIF: Professional identity formation
RToP: Ring Theory of Personhood
