# Supplementary material for: The impact of human cadaveric dissection on professional identity formation in medical students
Source: BMC Med Educ. 2023 Dec 19;23:970. doi: 10.1186/s12909-023-04913-x (PMC10731900; doi:10.1186/s12909-023-04913-x)
Supplement: Supplementary file 1 — Additional file 1. Survey Questionnaire [file 12909_2023_4913_MOESM1_ESM.pdf]

Do you consent to take part in this survey?

- ◯ Yes
- ◯ No

Yes

Demographics

Which gender do you identify as?

- ◯ Male
- ◯ Female

Age:

- ◯ 21 through 24
- ◯ 25 through 29
- ◯ Over 29

Please specify your ethnicity:

- ◯ Chinese
- ◯ Malay
- ◯ Indian
- ◯ __________ Others

Which of this best describes you?

- ◯ Singaporean
- ◯ Singaporean Permanent Resident

◯ Other

Entering year of Medical School:

Do you have a family member in your household who works as a doctor?

◯ No
◯ Yes

Motivation

Please rate yourself from "Not at all true of me" to "Very true of me" based on the statements below:

	Not at all true of me 1	2	3	4	5	6	Very true of me 7
I prefer course material that really challenges me so I can learn new things	◯	◯	◯	◯	◯	◯	◯
I prefer course material that arouses my curiosity, even if it is difficult to learn	◯	◯	◯	◯	◯	◯	◯
The most satisfying thing for me is trying to understand the content as thoroughly as possible	◯	◯	◯	◯	◯	◯	◯
When I have the opportunity, I choose course assignments that I can learn from even if they don't guarantee a good grade	◯	◯	◯	◯	◯	◯	◯
Getting good grades is the most satisfying thing for me right now	◯	◯	◯	◯	◯	◯	◯
The most important thing for me right now is improving my overall grade point average, so my main concern is getting good grades	◯	◯	◯	◯	◯	◯	◯
If I can, I want to get better grades than most of the other students	◯	◯	◯	◯	◯	◯	◯
I want to do well because it is important to show my ability to my family, friends, employer, or others	◯	◯	◯	◯	◯	◯	◯

Empathy

Please rate yourself from "Does not describe me well" to "Describes me very well" based on the statements below:

	Does not describe me well 1	2	3	4	Describes me very well 5
I often have tender, concerned feelings for people less fortunate than me	○	○	○	○	○
I sometimes try to understand my friends better by imagining how things look from their perspective	○	○	○	○	○
Other people's misfortunes do not usually disturb me a great deal	○	○	○	○	○
When I'm upset at someone, I usually try to "put myself in their shoes" for a while	○	○	○	○	○
I am often quite touched by things that I see happen	○	○	○	○	○
I try to look at everybody's side of a disagreement before I make a decision	○	○	○	○	○
Before criticizing somebody, I try to imagine how I would feel if I were in their place	○	○	○	○	○
I would describe myself as a pretty soft-hearted person	○	○	○	○	○

Dissection Experience

Please enter your hours for your dissection experience below:

Observed in-person human cadaveric dissection without hands-on participation for:
(minimum = 0 hours)

[ ]

Hands-on participation in human cadaveric dissection for:
(minimum = 0 hours)

[ ]

How well do the following statements describe you?

I believe that...

	Strongly disagree	Disagree	Agree	Strongly agree
human dissection is an essential part of learning medicine	○	○	○	○
human dissection makes me feel like I am a doctor	○	○	○	○

Mentor

How strongly do you agree with the following statements?

	Strongly disagree	Disagree	Agree	Strongly agree
I have a mentor in medical school with whom I have a personal connection	○	○	○	○
I have a mentor in medical school who advocates for me (e.g., seeks opportunities for me, or promotes me to others)	○	○	○	○

Learning Environment

Please indicate the category of frequency for each of the statement below:

	Never	Almost never	Some-times	Fairly often	Very often	Always
The educational experience makes students value themselves	○	○	○	○	○	○
The educational experience makes students feel a sense of achievement	○	○	○	○	○	○
The educational experience makes students feel confident of their academic abilities	○	○	○	○	○	○
There are disconnects between what I am taught about professional behaviours/attitudes and what I see being demonstrated by faculty	○	○	○	○	○	○
Faculty are helpful to students seeking advice not directly related to academic matter	○	○	○	○	○	○
When giving criticism or answering a question, faculty are genuinely interested in helping the student	○	○	○	○	○	○

	Never	Almost never	Some-times	Fairly often	Very often	Always
Faculty and administrators give personal help to students having academic difficulty	◯	◯	◯	◯	◯	◯
Faculty are reserved and distant with students	◯	◯	◯	◯	◯	◯

Professional Identity

Please indicate whether you agree or disagree with the following statements:

	Strongly agree 1	2	3	4	Strongly disagree 5
I feel like I am a member of this profession	◯	◯	◯	◯	◯
I feel I have strong ties with members of this profession	◯	◯	◯	◯	◯
I am often ashamed to admit that I am studying for this profession	◯	◯	◯	◯	◯
I find myself making excuses for belonging to this profession	◯	◯	◯	◯	◯
I try to hide that I am studying to be part of this profession	◯	◯	◯	◯	◯
I am pleased to belong to this profession	◯	◯	◯	◯	◯
i can identify positively with members of this profession	◯	◯	◯	◯	◯
Being a member of this profession is important to me	◯	◯	◯	◯	◯
I feel I share characteristics with other members of the profession	◯	◯	◯	◯	◯

Follow-up Interview

Do you consent to take part in the follow-up interview?

◯ Yes

◯ No

Interview Yes

Thank you for accepting the invite to the interview.

Please kindly fill up the following questions:

Name:	
Email:	
Age:	
Gender (M/F):	
Year of study:	
Human cadaferic dissection experience (Yes/No):	
